# Genetic Susceptibility to Chagas Disease: An Overview about the Infection and about the Association between Disease and the Immune Response Genes

**DOI:** 10.1155/2013/284729

**Published:** 2013-08-28

**Authors:** Christiane Maria Ayo, Márcia Machado de Oliveira Dalalio, Jeane Eliete Laguila Visentainer, Pâmela Guimarães Reis, Emília Ângela Sippert, Luciana Ribeiro Jarduli, Hugo Vicentin Alves, Ana Maria Sell

**Affiliations:** ^1^Program of Biosciences Applied to Pharmacy, Department of Clinical Analysis and Biomedicine, Maringa State University, Avenida Colombo 5790, 87020900 Maringa, PR, Brazil; ^2^Basic Health Sciences Department, Maringa State University, Avenida Colombo 5790, 87020900 Maringa, PR, Brazil; ^3^Departamento de Ciências Básicas da Saúde, Universidade Estadual de Maringá, Avenida Colombo 5790, 87020900 Maringa, PR, Brazil

## Abstract

Chagas disease, which is caused by the flagellate parasite *Trypanosoma cruzi*, affects 8–10 million people in Latin America. The disease is endemic and is characterised by acute and chronic phases that develop in the indeterminate, cardiac, and/or gastrointestinal forms. The immune response during human *T. cruzi* infection is not completely understood, despite its role in driving the development of distinct clinical manifestations of chronic infection. Polymorphisms in genes involved in the innate and specific immune response are being widely studied in order to clarify their possible role in the occurrence or severity of disease. Here we review the role of classic and nonclassic MHC, *KIR*, and cytokine host genetic factors on the infection by *T. cruzi* and the clinical course of Chagas disease.

## 1. Introduction

### 1.1. General Description of Chagas Disease

Chagas disease, or American trypanosomiasis, is an infection caused by the haemoflagellate protozoan *Trypanosoma cruzi*. It is one of the most important public health problems in Latin America and was first described by Carlos Justiniano Ribeiro das Chagas, a Brazilian physician and scientist [1]. The disease is endemic and is characterised by acute and chronic phases, which develop in the indeterminate, cardiac, and/or gastrointestinal forms. Their course of evolution may be influenced by both genetic and biological variability of the parasite and host [2, 3].

The World Health Organization estimates that approximately 10 million individuals are currently infected with *T*. *cruzi* with potential for developing cardiac or gut pathology normally associated with chronic Chagas disease [4] and a large fraction of them will die prematurely, usually from cardiac complications [5].

### 1.2. Origin and Discovery of Chagas Disease

The presence of *T. cruzi* is quite old, totalling about 100 million years. The historic evolution of the trypanosomes began from primitive aquatic invertebrates, and later in the digestive tract of vertebrates such as fish, amphibians, and reptiles. After that, haematophagous insect predators were able to transmit the parasite to different hosts that served as a food source, small to medium wild marsupial mammals, setting the enzootic cycle in the Americas. Thereafter, the cycle expanded to other mammals due to the behaviour of triatomines [6, 7]. 

The domestic cycle only settled down much later. The spread of disease is due to settlement and concentration of human populations in pre-Columbian times [8]. However, the establishment of Chagas disease itself as a zoonosis occurred 200–300 years ago, as a result of deforestation caused by the expansion of agriculture and livestock when humans approached the natural invertebrate niches [9]. 

There are indications that human infection with *T. cruzi* has occurred since at least nine thousand BC years in populations of the Andean countries; it was possible to identify molecular remnants of *T. cruzi* in mummies of these era and region [10], and Peruvian ceramics dating from the thirteenth to sixteenth centuries revealed possible representations of Chagas disease, including a head with unilateral ocular oedema, identical to the Romaña signal that often characterises the context of acute infection [11]. 

Charles Darwin observed the behaviour of the triatomine insect transmitter during his passage through Argentina and wrote in his diary “The Voyage of the Beagle” that he had been bitten by the same insect while visiting Chile in 1835. The presence of gastric symptoms and his final death caused by heart problems in 1882 was suggested to be due to Chagas disease [12]. 

The disease was described for the first time in 1907 when Carlos Chagas described the trypanosome, the transmission insect, and the syndrome that characterised a new tropical parasitic disease [1, 13].

### 1.3. Epidemiology

It is estimated that 10 million people are infected with *T. cruzi* worldwide, mostly in Latin America [4], and about 100 million people are at risk of the disease in the Americas, with a total estimated incidence of 800,000 new cases per year [14]. 

 Chagas disease was characterised as a neglected disease of poor and rural populations, but the progressive urbanisation, especially since the 1940, has made the disease an urban problem of medical and social importance. The disease has also spread from Latin America to nonendemic countries with the movements of people from endemic to nonendemic countries including North America, Western Pacific regions (mainly Australia and Japan), and Europe. It is estimated that today there are over 300,000 individuals infected with *T. cruzi* in the United States, over 5,500 in Canada, over 80,000 in Europe and the Western Pacific region, more than 3,000 in Japan, and more than 1,500 in Australia [15–18]. Thus, the prevalence, incidence, and mortality associated with Chagas disease showed considerable variations in recent decades, mainly due to the impact of control programs, migration of rural and urban populations, and socioeconomic changes [14]. Although the estimates of prevalence of infection are gradually decreasing, the disease still exists.

### 1.4. Transmission of Chagas Infection

The transmission of Chagas infection can be divided into primary and secondary mechanisms: the main mechanisms include transmission through insect vectors, by blood transfusion, contaminated food, and congenital transmission. Secondary mechanism transmissions may occur by laboratory accidents, organ transplants, sexual transmission, wounds in contact with contaminated sperm or menstrual fluid, and hypothetically by inoculation by criminal or deliberate contamination of food with the parasite [9]. 

The disease's reservoir lies in 100 different mammal species of wild animals. It is transmitted by several dozens of insect species belonging to the family Reduviidae, subfamily Triatominae. These insects hide in wild animals' nests or lairs and extract their blood meals (wild cycle). In the insect vector, the trypanosome undergoes several and successive developmental stages, terminating as a flagellated form that stays in the vector's rectum. At night, humans are bitten by these insects, usually in the facial area (domestic cycle). Ingestion of the blood meal causes the vector to defecate. After awakening, the victim usually rubs the bite area and pushes the stool with the trypanosome into the wound or onto the conjunctiva. After the *T. cruzi* accesses the victim's blood, this initiates the acute phase of the disease. Widely distributed via the blood stream, the trypanosome sheds its flagellum and penetrates tissue cells. They proliferate by binary fission within the cells (especially myocardium and meninges) [19].

Transfusion infection is the second most important epidemiological mechanism in the transmission of Chagas disease [19]. In 1960, the World Health Organization (WHO) estimated seven million cases per year due to blood transfusions in Latin America and this finding helped change policy and practice [14, 15]. Congenital transmission is transplacental and seems to depend on factors related to the parasite and the host; trypomastigotes penetrate the placenta through the chorionic epithelium and trophoblastic Hofbauer cells (macrophages placenta) where they transform into amastigotes [20]. Oral transmission of Chagas disease can occur, especially associated with contamination of breast milk (congenitally), fruit juices, and vegetables contaminated by infected wild vectors [21].

Other forms of exceptional transmission can occur. Accidents involve researchers and laboratory technicians working with the parasite through the blood of infected people and animals, culture media, or vector. The transmission by coitus has never been proven in humans; there are only reports of trypomastigotes in the blood of menstruation chagasic women. The presence of trypomastigotes was found in sperm from experimentally infected mice, and infection was also demonstrated after depositing *T. cruzi* in the vagina of rats [19]. 

### 1.5. Clinical Manifestations and Diagnosis

Human *T. cruzi* infection evolves from a usually oligosymptomatic acute phase to a chronic disease. The biological and genetic variability of the parasite and of the host may influence the course of disease progression [3, 22]. 

The early or acute phase of infection is characterised by high parasitaemia or trypomastigote circulating forms in the blood for two to four months [23]. During this period, the mortality ranges from 5% to 10% due to episodes of myocarditis and meningocefalite [24, 25]. The clinical signs associated with infection are a local inflammatory reaction with formation of strong swelling in the region of entry of the parasite (chagoma or Romaña sign), fever, splenomegaly, and cardiac arrhythmia [26]. The presence of circulating parasites can be detected by xenodiagnosis, haemoculture, [27] and molecular characterisation of the parasite's DNA by the polymerase chain reaction (PCR) [28]. During the acute phase, the majority of infected individuals develop a humoral and cellular immune response responsible for the decrease of parasites in the blood.

After that, the patients progress to the chronic asymptomatic stage that affects most individuals (50 to 60%); this condition characterises the indeterminate clinical form (IND) of the disease, and it may remain for long periods of time [23, 27]. About 20% to 30% develop cardiomyopathy that reflects a myocardium progressively damaged by extensive chronic inflammation and fibrosis and, in terminal phases, usually presents as dilated cardiomyopathy. Chronic Chagas cardiomyopathy (CCC) is the most relevant clinical manifestation causing death from heart failure in endemic countries and accounts for a significant burden of ischaemic and inflammatory heart diseases in the USA and Europe due to “globalisation” of Chagas disease. Eight to 10% has the digestive form, characterised by dilation of the oesophagus or colon (megaoesophagus and megacolon). Some patients have associated cardiac and digestive manifestations, known as the mixed or cardiodigestive form [17, 29, 30]. 

The transition from acute to chronic phase is accompanied by a marked decrease in parasitaemia, due to the mounting of a relatively effective immune response, which keeps parasite frequency at below detectable levels in the host. To diagnose the disease, regardless of stage, the serological test is used by detecting antibodies specific to the parasite: IgM (acute phase) and IgG (indeterminate and chronic phase) [31]. Conventional serological tests include primarily immunofluorescence assays (IFA), enzyme-linked immunosorbent assays (ELISA), and indirect haemagglutination assays (IHA). These tests were summarised by Afonso et al. [32]. Changes in the chronic phase can be revealed by electrocardiogram clinical diagnosis, X-rays, and ultrasound [27, 33].

### 1.6. Host Immune Response

There is a consensus that during *T. cruzi* infection the host immune system induces complex processes to ensure the control of parasite growth. The immune response is crucial for protection against disease; however, immunological imbalances can lead to heart and digestive tract lesions in chagasic patients. Several studies have evaluated the innate, cellular, and humoral immune responses in chagasic patients in an attempt to correlate immunological findings with clinical forms of Chagas disease. However, in all clinical forms of Chagas disease the involvement of cell-mediated immunity is undoubtedly of major importance [34–36].

It is also accepted that *T. cruzi* induces a strong activation of the immune system during acute infection and that the different immunological mechanisms triggered during the early indeterminate stages of the infection may represent an essential component of the immune activity observed during ongoing, clinically distinct chronic infection. After invasion of infectious metacyclic trypomastigotes in the mammalian host, *T. cruzi *infects a variety of cell types such as macrophages and fibroblasts. Fibroblasts are numerous in the extracellular matrix of the skin and are refractory to apoptosis [37] unlike macrophages and cardiomyocytes. Infected macrophages initiate the molecular interactions that mobilise the innate immune response of the host by secretion of proinflammatory cytokines like tumour necrosis factor alpha (TNF-*α*) and interleukin- (IL-) 12 [38]. These cytokines activate Natural Killer (NK) cells to produce interferon-gamma (IFN-*γ*) that acts directly on macrophages, activating them for antimicrobial activity. Activated macrophages produce nitric oxide (NO) via NO synthase (iNOS, NOS2), potent against *T. cruzi*. Furthermore, the regulatory cytokines—IL-4, IL-10, and transforming growth factor beta (TGF-*β*)—inhibit the production of NO, and trypanocidal activity of activated macrophages is responsible for disabling the control of lethal inflammatory effects of type 1 cytokines produced during infection [39].

Uncontrolled activation of NK cells and macrophages can lead to tissue damage. According to Vitelli-Avelar et al. [40], there is a mixed profile of cytokine production, and high levels of IFN-*γ*, TNF-*α*, and IL-4 favour the generation of inflammatory mechanisms. This intense inflammatory process during the initial infection is essential to confine the aetiologic agent in the intracellular site (limiting infection and symptoms) and prevent tissue damage but can be determinant to immunopathology. 

The lesions of the acute phase of the disease are characterised by the presence of localised inflammatory reactions, with a predominance of mononuclear cells at the foci of the pseudocyst ruptures, occasionally with the formation of granulomas located mainly in muscle and cardiac tissue [17].

There is a robust immune response displayed in the IND despite the complete lack of clinical disease. Many studies concerning the cellular immune response in the IND have been performed. Peripheral blood cells from these patients proliferate when stimulated with antiepimastigote antibodies [35]. Analysis of the expression of activation markers by T-cells showed that IND patients have a high frequency of CD4^+^ and CD8^+^ T-cells expressing HLA-DR and CD45RO [33]. Moreover, the vast majority of these T-cells do not express the costimulatory molecule CD28 [35] which suggested that this subpopulation displayed down-modulatory activity, due to intrinsic regulation via CTLA-4. In mice, deficiency in either class I- or class II-restricted T-cell populations was observed as a striking similarity in their mortality rate [41]. Given that CD8^+^ T-cells seem to be the best candidate for tissue destruction, it is possible that this regulatory mechanism, working in tandem with others, helps prevent pathology in IND [42]. 

Despite it being considered for decades that the adaptive immune response is the most important protective mechanism during chronic infection, recent studies have suggested the importance of the innate response as a regulatory mechanism for controlling morbidity during disease. Monocytes from IND patients, *in vitro*, lead to a high expression of IL-10, consistent with a modulatory response. CD3-D16^+^CD56^+^ and CD3^−^ CD16^+^ CD56^dim^ NK cells counts are particularly high, suggesting a protective role of this cell subpopulation. CD56^dim^ NK cells had more cytotoxic activity and can contribute to the control of parasitism. Added to this, asymptomatic patients exhibited Treg cells (CD4^+^CD25^high^), NKT regulatory cells (CD3^−^CD56^+^CD16^+^), and macrophage-like regulatory cells (CD14^+^CD16^+^) that encourage the establishment and maintenance of the IND form [39, 43].

CCC is associated with the presence of an intense inflammatory infiltrate in the myocardium, especially at sites where *T. cruzi* antigens are observed. The infiltrate was composed especially by CD8^+^ T-cells. IL-7 and IL-15 are critical for maintenance of these cells and their activation state in the heart tissue of CCC [44]. CD8^+^ T-cells are also found in the circulation. Similar to what was described in IND patients, CD8^+^ and CD4^+^ cells present high expression of HLA-DR and lower expression of CD28; however, here, CD4^+^ cells were correlated with the expression of TNF-alpha [35, 36] as well as IFN-*γ*. The latter cytokine is higher in CCC than IND patients and may be a key factor in the development of severe cardiomyopathy [45]. Monocytes from cardiac patients also produce TNF-*α* and IL-10. Although cells from CCC patients are able to produce IL-10, the ratio of this cytokine seems to be lower in cardiac patients [45].

During the *T. cruzi*-cardiomyocyte interaction, the parasite has control of the host cell gene expression, including expression of genes related to immune response, inflammation, cytoskeletal organisation, cell-cell and cell-matrix interactions, apoptosis, cell cycle, and oxidative stress [46]. The intense production of cytokines, chemokines, and nitric oxide that are essential elements of the defensive reaction in cardiac tissue can also result in cardiac hypertrophy. The activation of the host cell apoptotic machinery by pathogens is an offensive strategy to eliminate the host's immune response [47]. 

Megaoesophagus and megacolon are the major causes of morbidity in the digestive clinical form of chronic Chagas disease. Inflammatory infiltrates and fibrosis are found associated with lesions of muscle cells and of the intramural nervous system. They are composed mainly of CD3^+^CD4^+^ T lymphocytes, CD20^+^ B lymphocytes, CD57^+^ NK cells, and CD68^+^ macrophage-like cells [48]. Corrêa-Oliveira et al. [45] observed that patients with the gastrointestinal form of Chagas disease demonstrated a significant decrease in the absolute number of CD3^+^ T-cells as well as in CD19^+^ B lymphocytes, and an inversion of the CD4/CD8 ratio, contrasting with results from CCC where the ratio of these cells is normal. Chagasic patients with megacolon present increased numbers of eosinophils and mononuclear cells [49]. These cells are associated with inflammatory processes and can contribute to tissue injury through the secretion of cytokines such as IL-1, TNF-*α*, and IL-6, which activate the cytotoxic process [50].

### 1.7. Pathogenesis in Chagas Disease

Based on the relationship of parasite and host interaction, the mechanisms of pathogenesis in human Chagas disease can be based on two main hypotheses. The first defends the pivotal role of parasite's persistence in the host as a major cause of pathology, while the other postulates that an immune response against self-antigens is responsible for the tissue damage observed in affected organs of chagasic individuals. 

*T. cruzi* exhibits multiple strategies to ensure its establishment and persistence in the host. Although this parasite has the ability to infect different organs, heart impairment is the most frequent clinical manifestation of the disease. Calvet et al. [47] reviewed the current understanding of molecules involved in *T. cruzi*-cardiomyocyte recognition, the mechanism of invasion, and the effect of intracellular development of *T. cruzi *on the structural organisation and molecular response of the target cell. The nature of the myocardial changes in the chronic stage has been considered by some to be an autoimmune phenomenon based on antigenic mimicry in the form of an antibody targeting *T. cruzi *polypeptides [51]. More recently, however, persistence of the parasite in the tissues has been demonstrated [52]. 

Although the theories are controversial, autoreactivity and parasite persistence theories are not mutually exclusive. The variation in pathological manifestation has been reported and was related to differences in host immune response, such as the ability to control parasitaemia, the strength of inflammatory reactions, and the induction of autoimmune-like responses [53].

## 2. Genetic Factors and Their Influence on Chagas Disease

The advance in knowledge about infection and disease has changed the concept of infectious diseases, and the genetic markers play an important role in this area [54]. The spectrum of expression of Chagas disease brings strong evidence of the influence of the genetic factors on its clinical course [55].

The polymorphisms of the genes involved in the innate and specific immune response are being widely studied in order to clarify their possible role in the occurrence or severity of disease. To identify possible host genetic factors that may influence the clinical course of Chagas disease, the role of classic and nonclassic major histocompatibility complex (MHC) genes, killer cell immunoglobulin-like receptor (*KIR*) genes and cytokine genes, that are involved in the immune response will be addressed.

### 2.1. Strategy for Screening and Selecting Studies

**Figure 1:**
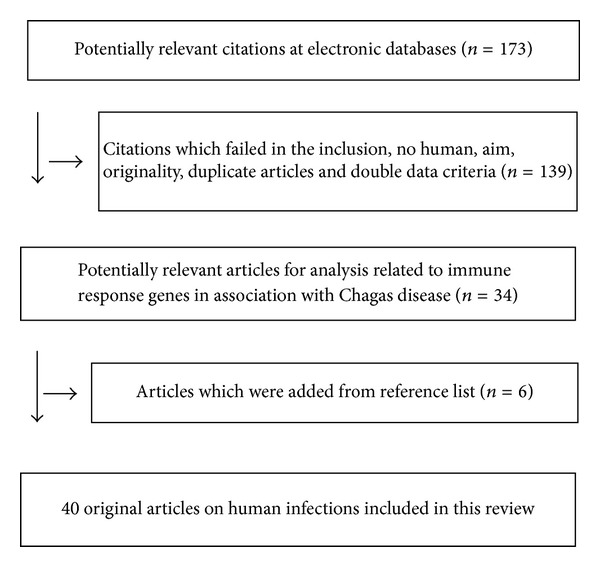
Flow chart of the study for review.

This review about genetic factors and their influence on Chagas disease selected original articles carried out on humans that were found in the databases of PubMed (US National Library of Medicine), LILACs (Latin American and Caribbean Center on Information in Health Sciences), and Google Scholar. The research period covered included the limit of databases until March 2013. There was no restriction regarding language. In the PubMed database MeSH (Medical Subject Heading Terms) were used, and in the LILACS descriptors were used. In order to retrieve articles of interest, free terms were used in the LILACS and Google Scholar. The MeSH terms, descriptors, and free terms were organized according to thematic groups: (i) HLA and Chagas disease (“Chagas Disease/genetics” OR “Chagas Disease/immunology” AND “HLA Antigens/genetics” OR “HLA antigens/immunology”); (ii) MIC and Chagas disease (“Chagas disease/genetics” AND “MHC class I-related chain A”); (iii) *KIR* genes and Chagas disease (“Chagas disease/genetics” AND “Receptors, *KIR*/genetics”); (iv) Cytokine genes and Chagas disease (“Chagas disease/genetics” AND “cytokines/genetics” OR “Chemokines/genetics” OR “Receptors, cytokine/genetics.” The immune response genes, as HLA, *KIR*, MIC, and cytokines, and their association with Chagas disease and its clinical forms in the American Latin population were presented. The results were summarized in Figure 1 and the selected studies are presented in Tables 1 and 2. 

### 2.2. HLA and Chagas Disease

To identify possible host genetic factors that may influence the clinical course of Chagas disease, the molecules and genes in the region of the human leucocyte antigen (HLA) have been analysed in patients presenting with differing clinical symptoms.

The highly polymorphic HLA class I (A, B, and C) and II (DR, DQ, and DP) molecules determine the efficiency of presentation of the *T. cruzi* epitopes to CD8^+^ and CD4^+^ T-cells, respectively. The type of the presentation could affect the clinical course of diseases because patients may respond differently to the same antigen, depending on their HLA repertory [56].

HLA alleles and haplotypes associated with Chagas disease are summarized in Table 1.

Several HLA alleles and haplotypes have been reported to be associated with Chagas disease. In Venezuela, a study comparing class II allele frequencies between patients and controls identified a decreased frequency of DRB1*14 and DQB1*03:03 in patients, suggesting independent protective effects to chronic infection in this population. In this same study, a higher frequency of DRB1*01, DRB1*08, and DQB1*05:01 and a decreased frequency of DRB1*15:01 were found in patients with arrhythmia and congestive heart failure [57]. In an endemic area of central Venezuela, a higher frequency of the HLA-DPB1*04:01 allele and DPB1*04:01-HLA-DPB1*23:01 or DPB1*04:01-DPB1*39:01 haplotype was found in patients with cardiac manifestations [58]. Susceptibility between HLA-C*03 and CCC Venezuelan was confirmed [59].

In Chile, HLA-B40 antigen in the presence of Cw3 was significantly lower in subjects with CCC [60] and was found in higher expression in patients without evidence of heart disease in Santiago [61]. 

An increase of HLA-A31, B39, and DR8 and a decrease of HLA-DR4, DR5, DQ1, and DQ3 were observed in several Latin American mestizos from different countries and with CCC [62]. 

A study in south-eastern Brazil showed that HLA-A*30 was involved in susceptibility to Chagas disease, whereas HLA-DQB1*06 was related to protection, regardless of the clinical form of the disease [63], and HLA-DR2 was associated with susceptibility to chronic Chagas disease in a south Brazilian population [64]. However, in another study, the polymorphism of HLA-DR and -DQ does not influence the susceptibility to different clinical forms of Chagas disease or the progression to severe Chagas' cardiomyopathy [65].

The haplotype HLA-DRB1*14-DQB1*03:01 was involved in resistance to infection with *T. cruzi* in a rural mestizo population of southern Peru [66].

In the Mexican population, HLA-DR4 and HLA-B39 were associated with the infection by the *T. Cruzi *whereas HLA-DR16 was a marker of susceptibility to damage to the heart and HLA-A68 was related with protection to development of CCC [67].

 In Argentina, the class II allele HLA-DRB1*04:09 and DRB1*15:03 was significantly more prevalent in Chagas disease and DRB1*11:03 allele was associated with disease resistance. Increased frequency of DRB1*15:03 allele was found among CCC suggesting susceptibility [68, 69].

In Bolivia, the frequencies of HLA-DRB1*01 and HLA-B*14:02 were significantly lower in patients suffering from megacolon, as well as in those with ECG alteration and/or megacolon, compared with a group of IND patients. The DRB1*01:02, B*14:02, and MICA*011 alleles were in strong linkage disequilibrium, and the HLA-DRB1*01-B*14-MICA*011 haplotype was associated with resistance against chronic Chagas disease [70].

These different results between the HLA allele and haplotypes and Chagas disease could be caused by variability of HLA allele's distribution in different ethnic groups; the typing test (serological or molecular techniques); the methods of statistical analyses (simple chi-square test and logistic or linear regression) and interpretation (*P* value or *P*_*c*_ value that applies the Bonferroni correction for multiple comparisons); the selection of the patients and the clinical form; the numbers of individuals; linkage disequilibrium; and biological variability of the parasite. Nevertheless, genetic factors related to the HLA system reflect an important role in susceptibility or protection to Chagas disease and its clinical forms. 

### 2.3. MIC and Chagas Disease

The HLA region contains not only classical HLA genes but also a wide variety of immunologically relevant genes, such as nonclassical class I genes (*MICA*, *MICB*; major histocompatibility complex class I chain-related genes A and B) that may be involved in disease pathogenesis.

The MICA molecules are recognised by lymphocytes T*γδ*, lymphocytes T*αβ* CD8+, and NK cells via their receptors NKG2D, present on their surfaces, in association with the DAP10 molecule, an adapter protein membrane [71, 72]. This complex, NKG2D-MICA, activates the phosphorylation of tyrosine residues of the molecule DAP10, triggering a cascade of cell signalling that ends with the process of cell lysis of the target cells [72]. A study by Steinle et al. [73] showed that changing a single amino acid—a methionine for a valine at position 129 of the *α*2 domain of the heavy chain—categorises the MICA as strong (MICA-129met) and weak (MICA-129val) ligands of NKG2D affecting the activation of NK cells.

MICA and MICB are weakly expressed on healthy cells, but their expression is induced in response to cellular stress in many cell types, including epithelial cells, fibroblasts, keratinocytes, endothelial cells, and monocytes [74–77]. 

The polymorphism of MICA may be involved in susceptibility to various diseases, but it has been suggested that this association may be secondary, due to the strong linkage disequilibrium with HLA-B alleles. Groh et al. [78] found that MICA*011, which was closely linked to HLA-B*14 and DRB1*01, might stimulate T*γδ* cells in the gut mucosa, a phenomenon that could relate to megacolon. In Chagas disease, the same HLA-DRB1*01-B*14-MICA*011 haplotype was associated with resistance against the chronic form [70]. *MICA-A5* and *HLA-B35* synergistically enhanced susceptibility to CCC [79]. 

### 2.4. Association of *KIR* Genes and Their HLA Ligands with Chagas Disease

Other receptors of NK cells that recognise HLA class I molecules present on the target cells included *KIR* (killer cell immunoglobulin-like receptor) [80, 81]. *KIR* receptors are glycoproteins that belong to the immunoglobulin superfamily, and which are also found in some subpopulations of T-cells [82]. *KIR* genes are clustered in the 19q13.4 region and are characterised by both allelic (high numbers of variants) and haplotypic (different numbers of genes for inhibitory and activating receptors on individual chromosomes) polymorphisms. The specific KIR-HLA combinations may regulate NK cell-mediated immunity against infectious pathogens and contribute to diverse susceptibility to diseases and other clinical situations.

Several studies have shown the participation of *KIR* genes and their ligands in infectious diseases [83–89]; autoimmune or inflammatory diseases [90–92], cancer [93–95], and in the success of transplantation [96]. However, there are no data available on the role of *KIR* genes in the immunopathogenesis of Chagas disease.

### 2.5. Association of Polymorphisms in Cytokine Genes with Chagas Disease

Immunomodulatory cytokines secreted by T-cells and macrophages are molecules that act as mediators of inflammation and immune response. These molecules are key components in the pathogenesis of many diseases including infectious diseases, cancer, metabolic disorders, autoimmunity, and inflammatory conditions. The cytokines are important for parasitic control and are involved in the genesis of lesions [97]. 

It is known that the production of some cytokines is under genetic control and is influenced by polymorphisms in several cytokine genes. SNPs or microsatellites mainly located in regulatory regions may affect gene transcription and cause interindividual variations. Some of these polymorphisms influence the level of cytokine production, which can confer flexibility in the immune response [98–101]. Thus, the presence of certain alleles may influence the course of bacterial and viral infections [102, 103] and confer susceptibility or resistance to autoimmune disease [104]. SNPs in cytokine genes have been described as important genetic factors in the occurrence of different clinical forms of Chagas disease. The genetic susceptibility of single nucleotide polymorphisms in cytokine genes to human Chagas disease was reviewed [105]. The cytokines polymorphisms associated with Chagas disease are summarized in Table 2. 

Proinflammatory cytokines play a key role in the development of CCC. Aiming to investigate the influence of the *TNF* polymorphisms on Chagas disease, *TNFA *(positions −308 rs1800629, −244 rs673, −238 rs361525, and −1031 rs1799964) and *TNFB* genotypes have been interrogated. No significant differences were observed with these alleles or haplotypes between patients and controls in a Peruvian population [106]. *TNF-308A* also was not associated with CCC in Brazilian patients [107]. However, the same SNP could be directly involved in the genetic susceptibility of the chronic phase and CCC in a Mexican population [108]. Another study, also in Brazilian patients, suggested that allele *TNF-238A*, which correlates with production of significantly higher levels of TNF-*α*, could influence the susceptibility to infection [109]. The *TNF-1031C and -308A* alleles were significantly associated with the development of CCC whereas the *TNF*-*1031TT* and *-308 GG* genotypes were associated with lower risk to develop CCC, in a Colombian population [110]. Regarding the *TNF* gene microsatellite, ten alleles were associated with Chagas disease in the Brazilian population: eight of them correlate with susceptibility (*TNFa2*, *TNFa7*, *TNFa8*, *TNFb2*, *TNFb4*, *TNFd5*, *TNFd7*, and *TNFe2*) and two with protection (*TNFb7* and *TNFd3*) against the development of the disease [111]. Previously, the occurrence of the *TNFa2 *microsatellite was correlated with reduced survival in severe cardiomyopathy [112]. Despite the controversial results, these data suggest the involvement of *TNF* in the course of Chagas disease.

Clinical, genetic, and epidemiological studies have linked lymphotoxin-*α* (LTA), a proinflammatory cytokine, to coronary artery disease and myocardial infarction. In Brazilian CCC patients, homozygosity with respect to the *LTA+80C* (rs2239704) and *LTA+252G *(rs909253) alleles was significantly more frequent and was associated with susceptibility, and the haplotype *LTA+80A+252A* was associated with protection against CCC. Furthermore, homozygosity for the *LTA+80A* allele correlated with the lowest levels of plasmatic TNF-*α* [113].

It appears that *IL6* gene polymorphism does not contribute to susceptibility in the clinical manifestations of Chagas disease. In a study in two independent populations of Colombia and Peru, no difference was observed for* IL6-174GC* (rs1800795) between Chagas disease and controls, or between asymptomatic patients and CCC [114]. 

Some *IL1B* alleles and haplotypes have been associated with susceptibility to inflammatory, autoimmune and infectious diseases. The *IL1B-511*(rs16944)*, IL1F10.3 *(rs3811058),* IL1RN.4 *(rs419598),* IL1RN 6/1 *(rs315952), and* IL-1RN 6/2 *(rs315951) polymorphisms were analysed in Chagas disease and *IL-1RN.4CC* genotype was clearly associated with *T. cruzi* infection and CCC development [115]. The *IL1B+5810G* (rs1143633) allele and the haplotype *IL1B-31* (rs1143627) *+3954* (rs1143634) *CT* were associated with an increased risk of CCC [116]. Therefore, these studies suggest that *IL1* gene cluster polymorphisms may play a relevant role in the susceptibility to development of CCC and that the effect of *IL1B* gene on chagasic cardiomyopathy predisposition is dose dependent [116].

IL-10 and INF-*γ* production by T cells promotes *T. cruzi* control and protects against fatal acute myocarditis [115–118]. The SNP *IL10-1082* (rs1800896)* A* allele, which is associated with a lower expression of IL-10, had higher frequency in CCC Brazilian patients when compared to the asymptomatic group [119]. However, in Colombian patients no differences in allele frequency and haplotype of the *IL10* gene were observed in symptomatic and asymptomatic patients [120]. Linkage disequilibrium analysis on microsatellite loci suggests epistasis between MHC and IL-10 on Chagas disease susceptibility/resistance [121]. The frequency of the *IFNG+874AA *(rs62559044), which is associated with reduced production of INF-*γ*, was increased in the Colombian patients suggesting that this SNP may be involved in susceptibility but not in the progression of Chagas disease [122]. 

IL-4 is described as a prototypical anti-inflammatory cytokine. It can modulate the host and parasite survivals that depend on a fine balance between Th1 responses: on one hand it will control parasitism and, on the other hand, enhance heart inflammation throughout the course of the infection [123]. In a Bolivian population, SNP *IL4-590 *(rs2243250) *T* allele was associated with protection against *T. cruzi* infection [124], although in another study only the *IL4RA *(IL-4 receptor-*α*; rs147781) *+148AA* genotype showed a weak association with the development of CCC [120].

TGF-*β* plays a pivotal role in Chagas disease, not only in the development of chagasic cardiomyopathy, but also in many stages of the *T. cruzi* life cycle and survival in the host cell environment through regulation of (i) parasite invasion of heart cells, (ii) an intracellular parasite cycle, (iii) inflammation and immune response, (iv) heart fibrosis and remodelling, and (v) gap junction modulation and heart conduction [125]. Several SNPs in the *TGFB1* gene that may affect cytokine production have been described. A significant difference in the distribution of the *TGFB1 10 *(rs1982073) *T* and *C* alleles between patients and healthy controls was observed in the Peruvian and Colombian populations: *TGFB1 10CC*, the high producer genotype, was increased in patients of both populations [126].

The IL-12 family of cytokines can influence Th17 and the production of IL-17 and INF-*γ*. The SNPs rs3212227 in the 3′UTR of the gene *IL12B* were investigated, and *IL12B 3*′*UTR  C* allele and *CC* genotype were significantly increased among CCC patients when compared to asymptomatic individuals [127].

*MIF* (*macrophage migration inhibitory factor*) −*173C* allele and *CC* genotype were related to the major risk of Chagas disease in the Colombian and Peruvian populations [128].

Some chemokines also had been associated with the development of CCC. *CXCL9 CC* (rs10336) and *CXCL10 GG* (rs3921) genotypes were less frequent and *CCR5 CC* (rs1799988) was more frequent in patients with left ventricular dysfunction, compared with patients without this dysfunction and may indicate that CXCL9 and CXCL10 are master regulators of myocardial inflammatory cell migration, perhaps affecting clinical progression to the life-threatening form of CCC [129]. Some SNPs of *CCR5 *gene were associated with the development of severe cardiomyopathy in an endemic area of Colombia, Peru, and Venezuela [130–132]. 

In the Brazilian population, *BAT1* (HLA-B associated transcript 1) *C* allele and *CC *genotype, at positions 22 C/G and −348 C/T, were associated with risk of cardiomyopathy [133]. In the same population, *MCP-1* (monocyte chemo-attractant protein-1) gene, *CCL2*-*2518A* allele (rs1024611), and the *CCL2-2518 AA* genotype correlate with susceptibility to chronic cardiomyopathy [134]. *BAT1*, *MCP-1*, and *LTA* may be involved in the pathogenesis of cardiac Chagas disease.

Despite the controversial results, these data suggest the involvement of cytokines in the course of Chagas disease. The balance between T_H_1/T_H_2/T regulatory cytokines has different and opposing influences on the likelihood of infection with *T. cruzi* and on the clinical course of the Chagas disease.

## 3. Concluding Remarks

Many genetic linkages and association studies have attempted to identify genetic variations that are involved in immunopathogenesis of Chagas disease. However, causal genetic variants underlying susceptibility remain unknown due to complexity of parasite and host. Susceptibility/resistance to Chagas disease involves multiple genetic variants functioning jointly, each with small or moderate effects. Immunological mechanisms protect against the disease but contribute to aggression and tissue damage. Genome wide association studies (GWAS) and next generation sequencing (NGS), at present lacking in Chagas disease, may help identifying novel genetic polymorphisms with genome scale associations.

The impact of pathogens on host cell functions, as well as genetic markers that were significantly associated with disease, was found on schistosomiasis [135], ascariasis [136], leprosy [137], tuberculosis [138, 139], malaria [140], dengue [141], hepatitis [142, 143], and HIV-1 [144–147]. In Chagas disease, the *2590T* allele (rs2243250) polymorphism in the promoter region of *IL4* gene is a marker for *IL4* haplotypes likely associated with protection against *T. cruzi* infection [124]. 

The identification of the specific genes influencing *T. cruzi* infection and Chagas disease through genome scans techniques could offer a particular opportunity for mapping genes of susceptibility or resistance. Further studies are necessary to clarify the relationship between genotype and development of disease and its clinical outcomes. 

The characterisation of the susceptibility genes and their variants has important implications, not only for a better understanding of disease pathogenesis, but also for the control and development of new therapeutic strategies for infectious diseases. 

## Figures and Tables

**Table 1:** Alleles and haplotypes HLA associated with Chagas disease.

HLA class I alleles and haplotypes	HLA class II alleles and haplotypes	Population	Clinical forms	Association	Reference
	^ a^ DRB1*01, DQB1*03:03	**Venezuela**	Chronic phase	Protection	[57]
	^ a^ DRB1*15:01		CCC	Protection	[57]
	^ a^ DRB1*08		CCC	Susceptibility	[57]
	^ a^ DRB1*01, DQB1*05:01		CCC	Susceptibility	[57, 58]
	^ a^ DPB1*04:01 ^a^ DPB1*04:01–*23:01 ^a^ DPB1*04:01–*39:01 haplotypes		CCC	Susceptibility	[58]
^ b^ C*03			CCC	Susceptibility	[59]

^ a^HLA-B40-Cw3 haplotype		**Chile**	CCC	Protection	[60, 61]

^ b^ HLA-A*30		**Brazil**	All clinical form	Susceptibility	[63]
	^ b^ DQB1*06		All clinical form	Protection	[63]
	^ b^DR2		Chronic phase	Susceptibility	[64]
				No association	[65]

	^ b^ DRB1*14-DQB1*03:01 haplotype	**Peru**	Infection	Protection	[66]

^ b^HLA-B39	^ b^HLA-DR4	**Mexico**	Infection	Susceptibility	[67]
^ b^HLA-B35	^ b^HLA-DR16		CCC	Susceptibility	
^ b^HLA-A68			CCC	Protection	

	^ b^ DRB1*04:09 and *15:03	**Argentina**	Infection	Susceptibility	[68, 69]
	^ b^ DRB1*11:03		Infection	Protection	[69]
	^ b^ DRB1*15:03		CCC	Susceptibility	[69]

^ b^ HLA-B*14:02	^ b^ HLA-DRB1*01	**Bolivian**	DG or mixed	Protection	[70]
	^ b^ HLA-DRB1*01-B*14-MICA*011 haplotype		Infection	Protection	[70]

^ b^HLA-B35-*MICA-A5* haplotype		**Guatemala**	CCC	Susceptibility	[79]

^ b^A31 and B39	^ b^DR8	**Latin American mestizos**	CCC	Susceptibility	[62]
	^ b^DR4, DR5, DQ1, DQ3		CCC	Protection	

^a^ *P* value ≤ 0.05 or ^b^ *P*_*c*_ value ≤ 0.05.

CCC: chronic Chagas cardiomyopathy; DG: digestive form; mixed: CCC and DG or CCC + DG.

**Table 2:** Polymorphisms in cytokines genes and their association with Chagas disease.

Gene/allele/genotype	Population	Clinical form	Association	Reference
*TNFA* −308, −244, −236 and *TNFB *	Peru	Infection and CCC	No association	[106]
^ b^ *TNF* −308*A *	Mexico	Infection and CCC	Susceptibility	[108]
*TNF* −308*A *	Brazil	CCC	No association	[107]
^ a^ *TNF* −238*A *	Brazil	Infection	Susceptibility	[109]
^ a^ *TNF* −*1031C *and −*308A *	Colombia	CCC	Susceptibility	[110]
^ a^ *TNFA* −*1031TT *and −*308GG *	Colombia	CCC	Protection	[110]
^ a^ *TNFa2, TNFa7, TNFa8, TNFb2, TNFb4, TNFd5, TNFd7, TNFe2 *	Brazil	CCC, DG, or mixed	Protection	[111]
^ a^ *TNFb7* and *TNFd3 *	Brazil	CCC and mixed	Susceptibility	[111]
^ a^ *TNFa2 *	Brazil	CCC	Susceptibility	[112]
^ b^ *LTA* +*80C* and *LTA* +*252G *	Brazil	CCC	Susceptibility	[113]
^ b^ *LTA* +*80A* +*252A *haplotype	Brazil	CCC	Protection	[113]
*IL6* −*174GC *	Peru/Colombia	Infection	No association	[114]
^ b^ *IL* −*1RN.4CC *	Mexico	CCC	Susceptibility	[115]
^ b^ *IL1B* +*5810G* allele and *IL1B* −*31* +*395 CT *genotype	Colombia	CCC	Susceptibility	[116]
^ a^ *IL10* −*1082A *and −*1082AA *	Brazil	CCC	Susceptibility	[119]
*IL10 *	Colombia	Infection	No association	[120]
^ a^ *IFNG* +*874A *and +*874AA *	Colombia	Infection	Susceptibility	[122]
^ a^ *IL4* −*590T *	Bolivia	Infection	Protection	[124]
^ a^ *IL4RA* +*148AA *		CCC	Susceptibility	[120]
^ a^ *TGFB110C *and *CC *	Peru/Colombia	Infection	Susceptibility	[126]
^ b^ *IL12B 3*′* UTR C *and *CC *	Colombia	CCC	Susceptibility	[127]
^ a^ *CXCL9CC *and *CXCL10GG *	Brazil	CCC	Protection	[129]
*CCR5CC *		CCC	Susceptibility	[129]
^ a,b^ *CCR5* −*2554T, *−*2733G, 59029G, 59029AG, 59029GG *	Peru/Colombia/Venezuela	CCC	Susceptibility	[130–132]
^ a^ *CCL2* −*2518A *and *AA *	Brazil	CCC	Susceptibility	[133]
^ a^ *BAT1 22C 348C *	Brazil	CCC	Susceptibility	[134]

^a^ *P* value ≤ 0.05; ^b^ *P*_*c*_ value ≤ 0.05.

CCC: chronic Chagas cardiomyopathy; DG: digestive form; mixed: CCC and DG or CCC + DG.
